# Variations and Transmission of QTL Alleles for Yield and Fiber Qualities in Upland Cotton Cultivars Developed in China

**DOI:** 10.1371/journal.pone.0057220

**Published:** 2013-02-27

**Authors:** Tianzhen Zhang, Neng Qian, Xiefei Zhu, Hong Chen, Sen Wang, Hongxian Mei, Yuanming Zhang

**Affiliations:** 1 National Key Laboratory of Crop Genetics & Germplasm Enhancement, MOE Hybrid Cotton R&D Engineering Research Center, Nanjing Agricultural University, Nanjing, People’s Republic of China; 2 Cotton Research Institute, Xinjiang Academy of Agriculture and Reclamation Sciences, Xinjiang, People’s Republic of China; New Mexico State University, United States of America

## Abstract

Cotton is the world’s leading cash crop, and genetic improvement of fiber yield and quality is the primary objective of cotton breeding program. In this study, we used various approaches to identify QTLs related to fiber yield and quality. Firstly, we constructed a four-way cross (4WC) mapping population with four base core cultivars, Stoneville 2B, Foster 6, Deltapine 15 and Zhongmiansuo No.7 (CRI 7), as parents in Chinese cotton breeding history and identified 83 QTLs for 11 agronomic and fiber quality traits. Secondly, association mapping of agronomical and fiber quality traits was based on 121 simple sequence repeat (SSR) markers using a general linear model (GLM). For this, 81 *Gossypium hirsutum* L. accessions including the four core parents and their derived cultivars were grown in seven diverse environments. Using these approaches, we successfully identified 180 QTLs significantly associated with agronomic and fiber quality traits. Among them were 66 QTLs that were identified *via* linkage disequilibrium (LD) and 4WC family-based linkage (FBL) mapping and by previously published family-based linkage (FBL) mapping in modern Chinese cotton cultivars. Twenty eight and 44 consistent QTLs were identified by 4WC and LD mapping, and by FBL and LD mapping methods, respectively. Furthermore, transmission and variation of QTL-alleles mapped by LD association in the three breeding periods revealed that some could be detected in almost all Chinese cotton cultivars, suggesting their stable transmission and some identified only in the four base cultivars and not in the modern cultivars, suggesting they were missed in conventional breeding. These results will be useful to conduct genomics-assisted breeding effectively using these existing and novel QTL alleles to improve yield and fiber qualities in cotton.

## Introduction

Cotton is the most important natural textile fiber source globally. The worldwide economic impact of the cotton industry is estimated to approximately $500 billion per year with an annual utilization of approximately 115 million bales or 27 million metric tons of cotton fiber. The tetraploid species, *Gossypium hirsutum* L. (n = 26, AD genome), also referred to as ‘Upland cotton’, accounts for 95% of the world’s cotton production (National Cotton Council, USA, http://www.cotton.org, 2006). Current and obsolete cultivars of Upland cotton have been the main sources of cotton breeding program worldwide.

China is the largest cotton-growing nation, but is not an Upland cotton domestication country. Most cotton cultivars planted in China were derived from a few sources of germplasm such as Deltapine (DPL), Stoneville (STV), Foster and King, all of which were introduced from America. These cultivars represent the foundation of Chinese cotton breeding program and played a crucial role in the development of Chinese self-breeding cultivars. Cotton breeding in China has experienced several periods and cotton cultivar replacement began initially in the 1920’s. In 1919, the King cultivar was introduced and followed by Trice and Lonestar cultivars in 1920. STV 4 and Delfos 531 were introduced in 1935–1936 and DLP in 1946. In 1950, large quantities of DPL 15 and STV 2B were introduced directly to replace all *G. arboretum* cultivars planted in China for several thousands of years and those deteriorated Upland cotton cultivars which were previously introduced [1]–[2]. In 1959, several cultivars were developed from cotton introduced from Uganda. Following their introduction, Chinese breeders started to develop cultivars *via* pedigree selection (PSP), and later hybridization programs (HSP) were conducted to develop high yield cotton cultivars with resistance to Fusarium wilt [2]. Therefore, the genetic base was narrow and, as a result, the genetic diversity of Upland cotton was low, especially in China due to the limited quantity of sources used [3]–[4].

Intra-specific genetic linkage maps of Upland cotton have been developed and used to identify quantitative trait loci (QTL) for agronomy and fiber quality traits [5]–[15]. The use of linkage disequilibrium (LD)-based association mapping has been suggested as a powerful genetic tool to identify DNA markers that are in LD with a locus controlling the trait of interest. This method is convenient because it helps avoiding the need to screen large bi-parental mapping populations [16]. LD can be detected statistically, and has been used to map genes underlying complex genetic traits in humans [17]–[18]. Association mapping was introduced to plant genetics in 2001 [19] and was subsequently applied to many plant species [20]–[21]. Identification of QTL by association mapping is widely used and has been employed in genetic studies of rice, corn, barley and other important agricultural crops [22]–[25]. Breseghello et al. (2006) used association mapping in 95 cultivars of soft winter wheat to identify alleles for kernel size and milling quality [26]. On the basis of association of 62 SSR loci with kernel size and milling quality traits, the authors compared the average phenotypic value of accessions with specific alleles and null alleles, and were able to identify several alleles potentially beneficial for these traits. In this study ‘null allele’ referred to markers which were no longer detected by PCR because of a mutation. Therefore, the phenotypic effect was judged by the marker’s other allele [27]. However, not all of the markers may have null alleles, and even if they exist, they may be difficult to identify.

In the present study, four previously introduced Upland cotton cultivars (STV 2B, Foster 6, DPL 15, and CRI 7, a Ugandan germplasm-derived cultivar) were used to construct a four-way cross mapping population (4WC). This population was used to detect QTLs influencing agronomic and fiber quality traits. At the same time, we conducted LD-based association mapping using simple sequence repeat (SSR) markers. We measured important agronomic and fiber quality traits in 81 representative cultivars that were cultivated in China before transgenic cotton was introduced. Using this approach, a draft transmission table of QTLs and QTL alleles of breeding traits in Chinese Upland cotton was obtained. Some elite QTL alleles for yield and fiber quality traits were mined. The results provide preliminary insight into the genetic basis and diversity of Upland cotton cultivars, and offer useful information for cotton breeding and for further research.

## Materials and Methods

### 4WC Mapping Population and Trait Evaluation

STV 2B, Foster 6, DPL 15 and CRI 7 seeds were made available from the Cotton Research Institute, Chinese Academy of Agricultural Sciences (CRI-CAAS). A mapping population consisting of 239 individuals was constructed from the 4WC (STV 2B/Foster 6//DPL 15/CRI 7), grown and evaluated for fiber quality and yield in 2007 in the Jiangpu Breeding Station of the Nanjing Agricultural University (JBS/NAU), Nanjing, China. Due to a lack of enough self-pollinated seeds, 220 4WC families (F_2∶3_ progeny families) were grown in 2008 in JBS/NAU in one-row plots with a randomized block design in triplicates to evaluate their performance. The plot was 0.8 m wide and 5 m long and the plant density approximated 37,500 plants ha-1. Fifteen individuals per replication were measured and averaged (n = 3) for each trait from the four parents and 220 4WC families in 2008. The following seven agronomic traits were evaluated: Plant height (PH, cm), number of fruit branches per plant (PB), number of bolls per plant (NB), boll weight (BW, g), lint percentage (LP), lint index (LI) and seed index (SI). Lint yield (LY) was determined by multiplying lint percentage with total seed cotton weight. The following fiber quality traits were evaluated by HVI spectrum: 2.5% fiber span length (FL, mm), strength (FS, cN/tex), elongation (FE), micronaire reading (FM), and uniformity ratio (FU).

### Linkage Map Construction and QTL Mapping

DNA was extracted from 239 4WC individuals, two F_1_s and the four inbred parents as described before in our laboratory [28]. To screen for polymorphisms among inbred lines parents, 8,342 SSR primer pairs available in our laboratory were used. These SSRs included NAU, BNL, CIR, JSEPR, STV, MUSS, MUCS, TM, CER, CGR, DC, DPL and SHIN, which were described previously in detail [29]–[34]. Primers sequences can be obtained from Cotton Microsatellite Database (CMD, http://www.cottonmarker.org). Marker nomenclature consisted of a letter that specified the origin of the marker, followed by the primer number. The procedure for SSR analysis followed our published method of Zhang et al.(2000) [35].

All SSR primer pairs were used to screen for polymorphisms among STV 2B, Foster 6, DPL 15 and CRI 7. If one locus screened for polymorphisms was homozygous in two of the F_1_ parents (aa_bb), this locus would be excluded from linkage analysis because the alleles would not segregate in 4WC. The polymorphic markers identified between STV 2B and Foster 6, or DPL 15 and CRI7 were used to survey 239 individuals of the 4WC. A Chi-square test for goodness of fit was used to assess Mendelian segregation ratios, including 1∶1, 1∶2:1, 3∶1 and 1∶1:1∶1 ratios in 4WC.

JoinMap 3.0 [36] was employed to construct linkage maps, and linkage groups were assigned to chromosomes based on anchored markers in a high dense linkage map [28].

QTL analysis was carried out using the program Map-QTL 5.0 [37]. The significance thresholds for LOD scores were calculated by permutation tests in Map-QTL 5.0, with a genome-wide significance level of α = 0.05, n = 1,000 as significant QTL and a linkage group-wide significance level of α = 0.05, n = 1,000 as suggestive QTL [38]. QTL position indicated location of the peak. QTL nomenclature was adapted according to the method in rice [39], starting with ‘q’, followed by an abbreviation of the trait name (for example FL for fiber length, FS for fiber strength, etc.) and the name of chromosome, then followed by the number of QTL affecting the trait on the chromosome.

### Population-based Association Mapping

A total of 81 representative Upland cotton cultivars were used in this experiment (**Table 1**). These cultivars (excluding transgenic Bt cotton) were made available from the cotton germplasm collection in our laboratory and CRI-CAAS. These can be grouped into three types as follows: the first type includes those cultivars directly introduced and planted from USA and Uganda, the second type includes improved cultivars developed using PSP or once HSP from the first type cultivars, and the third type includes further improved cultivars developed with HSP or other breeding methods. Furthermore, these cultivars can be still classified on the basis of their ecological areas: the Yangtze River valley, the Yellow River valley, the Northern China area and America (**Table 1**).

**Table 1:** 81 Upland cotton cultivars used in the present research.

No.	Name	Ecological area	Pedigree	Introduced or authorized year	Breeding period
1	King	America	King	1890	1
2	Foster 6	America	Foster	1933	1
3	STV 2B	America	Stoneville	1947	1
4	DPL15	America	DPL14 pedigrees	1950	1
5	DPL16	America	DPL15 pedigrees×DPL45	1970	2
6	TM-1	America	DPL14 pedigrees		/
7	MD51ne	America	DP90*3×MD65-11ne	1991	/
8	Jimian 7	Yellow river	(Shanmian4×Shanmian6) ×Ji75-23	1980	2
9	Yumian 1	Yellow river	Shanmian4×Liuzhuang1	1981	3
10	Lumian 1	Yellow river	(CRI2×DPL15 pedigrees) induced	1979	2
11	Lumian 4	Yellow river	DPL16×Uganda4	1983	3
12	Shanmian 4	Yellow river	CRI3(57–681+Liaomian2)	1970	2
13	CRI 3	Yellow river	DPL15 pedigrees	1960	2
14	CRI 17	Yellow river	[(Zhong3723×Shan401) (Xuzhou209×910и)] CRI10	1990	3
15	CRI 19	Yellow river	CRI17[(CRI4×Shan5012) (Uganda4×Jimian1)]	1992	3
16	CRI 34	Yellow river	{[CRI2× (209+Jinkui)] × (Uganda3+Shan3778)} ×DPL45	1998	3
17	CRI 35	Yellow river	[(Shanmian4×TangGrace) (Xinongzhong3+CRI10+CRI16)] (Zhong12×Chuan174)	1999	3
18	Yumian 9	Yellow river	(Shanmian7×Xingtai6871) ×CRI10	1994	3
19	Shanmian 7	Yellow river	[(Pengze1×Xuzhou209) × (52–128+Xuzhou1818+60-5)] ×57–681	1970	2
20	Yumian 5	Yellow river	CRI10× (Heishan1×Mianxiang1)	1989	3
21	Sumian 5	Yellow river	Liaomian7×Shan274	1993	3
22	CRI 16	Yellow river	CRI10×Liao4086	1990	3
23	Shiduan 5	Yellow river	Coker100 pedigrees	1950	1
24	Keyi 2	Yellow river	Shiduan5×Huadong58-6635	1970	2
25	Jimian 1	Yellow river	Xuzhou1818 pedigrees	1975	2
26	Jimian 8	Yellow river	[Jihan5× (DPL45A×Zihuamian)] induced	1983	3
27	Jimian 12	Yellow river	Jihan5×Jimian1	1986	3
28	Shiyuan 321	Yellow river	{86-1× [(Giza×C.thurberi)F2 ×ASIT-2]F3}×Zhong12	1998	3
29	Xuzhou 1818	Yellow river	Xuzhou209 pedigrees	1961	2
30	Xuzhou 142	Yellow river	Xuzhou58 pedigrees	1973	2
31	Lumian 2	Yellow river	Jimian1×SP21	1983	3
32	Lumian 6	Yellow river	Jimian1×114	1984	3
33	Jingsimian	Yellow river	Stoneville 4 pedigrees	1942	1
34	Shanmian 1	Yellow river	Si517×Coker100	1957	2
35	86-1	Yellow river	[(55–90×DPL15) (Xuzhou209+Daifumian)](Ganmian1×Xuzhou209)	1990	3
36	CRI 23	Yellow river	{[(5658×Shan5245) ×4067] CRI10}Jimian8	1995	3
37	CRI 41	Yellow river	CRI23 integrated with Bt and CpTI genes	2002	3
38	CRI 5	Yellow river	Xuzhou209× (DPL15+Zijinkui)	Unknown	3
39	Difenmain8	Yellow river	Unknown (one parent of Wanza 40 hybrid cotton)	Unknown	3
40	CRI 45	Yellow river	95-1 integrated with Bt and CpTI genes	2003	3
41	CRI 4133	Yellow river	Unknown (one parent of CRI 28 40 hybrid cotton)	Unknown	3
42	Yumian 21	Yellow river	CRI12×Yu177	1999	3
43	Xuzhou 514	Yellow river	CRI7×Xuzhou142	1990	3
44	Lumian 5	Yellow river	Uganda3×Jimian1	1983	3
45	Shan 6192	Yellow river	(CRI7×DPL16) [(Emian6×Eguangmian) Jimian1]	Unknown	3
46	CRI 7	Yellow river	Uganda3	1959	2
47	CRI 12	Yellow river	Uganda4×Xingtai6871	1989	3
48	86-6	Yellow river	CRI12(DPL16×86-1)	Unknown	3
49	CRI 15	Yellow river	CRI12×36-1	Unknown	3
50	Shan1155	Yellow river	Zhongchang1×Xuzhou1818	1978	2
51	Yuanmian2	Yangtze river	(Naked leaf DPL×Yuan1127) Daihongdai	1970	2
52	Esha28	Yangtze river	(Jingmian4×Gangmian1) Jingmian4	1990	3
53	Hua101	Yangtze river	Esha28×DPL80	1992	3
54	Daihongdai	Yangtze river	(DPL15×Yishuhong) DPL15	1970	2
55	Xiangmian16	Yangtze river	(Dai27×86-1)F_4_× (Wufen1×Lambright low gossypol)F_4_	1994	3
56	Dongting1	Yangtze river	DPL15 pedigrees	1950	1
57	Xiangmian10	Yangtze river	(Daihongdai×Shanmian4) ×Daihongdai	1980	2
58	Simian2	Yangtze river	Dongting1 pedigrees×Mexico910	1989	3
59	Sumian3	Yangtze river	(Shanmian4×Liuzhuang1) Tong77-369	1990	3
60	Sumian6	Yangtze river	(Shanmian4×Shanmian6) AC241	1994	3
61	Sumian9	Yangtze river	Simian2×CRI12	1995	3
62	57-681	Yangtze river	DPL15 pedigrees	1963	2
63	Chuan414	Yangtze river	Zhong364×Shan401	1986	3
64	Yanmain1	Yangtze river	Changza1[(Changza1×Chuan73-27) Chuan73-27]	1987	3
65	Chuanmain56	Yangtze river	(Chuan473A×Jiangsu203) Shan1155	1992	3
66	Simian3	Yangtze river	{Simian1[(Pengze1×Xuzhou209) 52–128]}(Xuzhou58×CRI7)	1993	3
67	Simian4	Yangtze river	Simian3×Nantong84-239	2000	3
68	I40007	Yangtze river	Simian 3 pedigrees(one parent of Wanza 40 hybrid cotton)	Unknown	3
69	52–128	Yangtze river	Delfos531 pedigrees	1950	1
70	Ejing1	Yangtze river	(Jinmian2×Jingmian4) AntongSP-21	1992	3
71	Ejing92	Yangtze river	Jinmian2×Jingmian4	1980	2
72	Emian14	Yangtze river	Jimian1 pedigrees × CRI7	1990	3
73	Sumian1	Yangtze river	86-1[(Daihongdai × DPL16) Heishanmian1]	1988	3
74	Yanmian48	Yangtze river	86-1× Lumian1	1990	3
75	Sumain12	Yangtze river	(8004× Jihe328)× Jiangsu9101	1997	3
76	Sumian16	Yangtze river	Liaomian3(Putaomian×Chuanjian6)	1999	3
77	Ganmian8	Yangtze river	(Xuzhou142×Liaomian3) pedigrees	1991	3
78	XiangSC-24	Yangtze river	Unknown	Unknown	Unknown
79	8891	Yangtze river	Unknown	Unknown	Unknown
80	Sumian2	Yangtze river	(CRI7×DPL15) [(Xuzhou142×Shannong350) Jimian1]	1989	3
81	Liaomian4	Northern China	(Liaoyangduanjie×Stoneville2B) [(Guannong1×Stoneville2B)+Liaoyang1]	1972	2

Eighty one cultivars were grown and evaluated in three locations: JBS/NAU in the Yangtze River valley cotton growing region from 2006 to 2008; Linqing/Shandong in the Yellow River valley cotton growing region in 2008, Kuerl/Xinjiang in 2007 in the Northwestern cotton growing region, and Sanya/Hainan in the Southern cotton growing region during 2007 and 2008. A completely randomized block design with duplicates was employed for the field trials. The field management was adjusted to local practice. The same 12 agronomic and fiber quality traits mentioned above (see 4WC mapping populations) were evaluated.

The genome-wide LD between pairs of SSR marker loci was studied according to Witt and Buckler (2003) using the software package TASSEL ver. 2.0i (http://www.maizegenetics.net/tassel) [40]. LD was estimated by a weighted average of squared allele frequency correlations (r^2^) between SSR loci. The significance of pairwise LD (p-values≤0.01) among all possible SSR loci was evaluated using TASSEL with the rapid permutation test using 10,000 random draws with replacement. The LD values between all pairs of SSR loci were plotted as triangle LD plots using TASSEL to estimate the general view of genome-wide LD patterns and evaluate LD structures. The r^2^ values for pairs of SSR loci were plotted as a function of map distances (cM), and LD decay (at r^2^<0.1) was estimated [40].

To evaluate the population structure of the association mapping population, the software package STRUCTURE 2.2 [41]–[43] was employed to subdivide cultivars into genetic subgroups. One hundred thirty one unlinked or distantly linked marker loci (hereafter referred to as ‘‘unlinked’’), distributed over all the cotton chromosomes, were used for assessment of population structure. The number of subgroups (K) was set from 1 to 10. For each K, three runs were performed separately. The burn-in was set to 10,000 and the number of replications was set to 100,000.

The general linear model (GLM) association test was performed according to Yu et al.(2006) [44] using the TASSEL software package [45]. The agronomic and fiber quality traits from the seven environments, JBS/NAU in 2006 (06JS), 2007 (07JS) and 2008 (08JS), Xinjiang in 2007 (07XJ) and 2008 (08XJ), Hainan in 2008 (08HN), Shandong in 2008 (08SD), and the average data of seven environments (AV) were used, incorporating population structure information (Q matrices) as a covariate and using 1,000 permutations for the correction of multiple testing.

### DNA Extraction and Microsatellite Markers

An equal quantity of fresh, young leaves from each variety were collected and immediately brought to the laboratory where total genomic DNA was extracted as described before in our laboratory [28].

Our study is based on a genetic map which contains 3,147 loci in 26 linkage groups and was constructed in our laboratory [28], [46]. We selected one pair of SSR primers every 10 cM on this map. This resulted in use of 402 primer pairs to screen the 81 cultivars and to ensure a broad genome-wide coverage of genotyping and a representative estimation of genetic distances.

### Mining of QTL Alleles

Based on results of SSR association with the 12 traits, QTL alleles that associated significantly with the traits were further analyzed. The phenotypic allele effect was estimated through comparison between the average phenotypic value over accessions with the specific allele and that of all accessions:
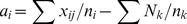
where *a_i_* is the phenotypic effect of the *i*th allele; *x_ij_* is the phenotypic value over the *j*th material with the *i*th allele; *n_i_* is the number of materials with the *i*th allele; *N_k_* is the phenotypic value over all accessions; *n_k_* is the number of all accessions. If *a_i_*>0, it is supposed to be the positive allele, if it is <0, it corresponds to the negative allele.

## Results

### 4WC and Family-based QTL Mapping for Yield and Fiber Qualities

Mean values, standard deviation, ranges, skewness, and kurtosis for traits measured in the parents and 4WC families are shown in **Table 2**. All traits from these four data sets exhibited continuous distribution in the 4WC population. ANOVA showed that there were significant differences (P<0.05) for all 12 traits among the four parents and in the population tested here.

**Table 2:** Phenotypic variation of traits in four parents and their F_2∶3_ families.

Traits^a^	Parent lines				F_2∶3_ Family Lines				
	Stoneville2B	Foster 6	DPL15	CRI7	Mean	Min	Max	Skewness	Kurtosis
PH	87.80	100.90	86.80	93.10	99.29±6.27	75.00	114.00	−0.08	0.21
PB	13.70	16.40	14.60	14.80	15.31±1.58	11.20	20.00	0.02	0.01
BW	4.77	4.94	5.52	5.02	5.68±0.45	4.40	7.01	0.05	−0.01
NB	12.00	11.40	10.70	10.40	11.91±2.56	6.00	21.40	0.34	0.22
LP	38.02	33.54	41.88	33.16	35.63±0.02	31.90	42.51	0.73	1.64
SI	12.06	11.65	11.00	11.25	11.57±0.71	9.38	13.62	0.05	0.02
LI	7.40	5.88	7.93	5.58	6.40±0.46	5.25	7.68	0.08	−0.10
FL	28.42	26.87	29.08	29.02	28.35±0.88	26.01	30.74	0.09	−0.43
FS	28.60	29.10	31.65	29.15	30.18±1.49	26.15	35.20	0.40	0.28
FM	5.40	5.05	4.75	4.80	4.92±0.35	4.15	6.50	0.99	3.28
FU(%)	82.85	84.10	81.20	83.55	83.52±1.02	80.80	88.10	0.46	1.33
FE(%)	5.85	5.95	4.90	5.45	5.70±0.53	4.35	7.15	0.17	0.29

a Plant height (PH), No. fruit branches per plant (PB), No. bolls per plant (NB), boll weight (BW), lint percent (LP), lint index (LI), seed index (SI), fiber length (FL), fiber strength (FS), micronaire reading (FM), fiber elongation (FE), and fiber uniformity ratio (FU).

**Figure 1:**
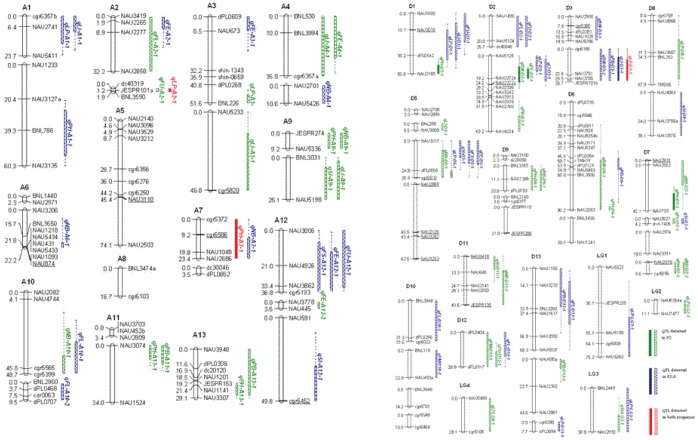
Location for QTL associated with yield and fiber quality traits in the population derived from the 4WC of STV 2B/Foster 6//DPL15/CRI 7 in Upland cotton. Positions of loci are given in centi-Morgans. Bars and lines indicate one LOD (tenfold) and two LOD (100-fold) likelihood intervals. The solid bars and lines indicate significant QTLs, and empty bars and dashed bars are suggestive of QTLs. Eighty-three QTLs are shown as plant height (PH), plant branches (PB), number of bolls per plant (NB), boll weight (BW), seed index (SI), lint percent (LP), lint index (LI), fiber length (FL), fiber strength (FS), Micronaire reading (FM), fiber elongation (FE), and fiber uniformity ratio (FU). __ Indicates distorted markers.

Of 8,324 SSR primers, only 238 (2.85%) detected polymorphisms between STV 2B and Foster 6, and DPL 15 and CRI 7, and generated 246 loci. In this 4WC screening, three polymorphic types comprising two, three and four alleles can be theoretically identified. Out of the 246 polymorphic loci, 240 (97.6%) produced two alleles, 6 (2.4%) three alleles (ab_ac), but none produced four alleles at one locus (ab_cd). A linkage map with 201 SSR loci and 58 linkage groups was constructed, and covered a length of 1691.0 cM with an average interval of 8.4 cM between loci. Based on our microsatellite-based, gene-rich linkage map [28], [46], 54 linkage groups were assigned to 25 chromosomes except chromosome D4 (chro.D4), in which 24 linkage groups assigned to the A-subgenome (which contained 86 loci and spanned 654.3 cM) and 30 linkage groups assigned to the D-subgenome (containing 104 loci and spanning 885.4 cM) (**Figure 1**).

The data for yield and fiber qualities of 239 4WC-F_2_ plants and their 220 F_2∶3_ family lines were used to detect QTLs by interval mapping. As a whole, 83 QTLs were identified which explained 2.6% to 73.9% of the total phenotypic variance (PV). A summary of characteristics of the QTLs detected in each analysis, including position, confidence interval, LOD score, the mean value of four different genotypes, PV, additive effects of a_1_ and a_2_ and overall dominance effect (d) are shown in Figure 1 and Table S1. A total of 59 QTLs for yield components and 24 QTLs for five fiber qualities were detected in two progenies. Among 59 QTLs for yield components, seven (qPH-A7-1, qPH-D1-1, qPH-D7-1, qBW-D2-1, qLP-A2-1, qLP-D3-1 and qLI-D3-1) were significant, and three were detected in both generations. In the significant QTLs contributing to PH, qPH-A7-1 with minus a_1_ meant that the synergistic site came from Foster 6. Similarly, both qPH-D1-1 and qPH-D7-1 came from STV 2B (positive a_1_) and DPL 15 (positive a_2_), qBW-D2-1 from Foster 6 and DPL15, and qLI-D3-1 from STV 2B and DPL15. Accordingly, compared with the other three parents, DPL15 had a higher impact on agronomic traits. Among the 24 QTLs for the five fiber qualities detected, STV 2B contributed six QTLs which led to an increase in FM and FL, Foster 6 contributed nine QTLs leading to an increase in FL, FS and FU, and DPL15 and CRI7 contributed eight and seven QTLs, respectively, to enhance the fiber qualities.

Comparing 4WC QTL mapping with our previously published results using the traditional family-based linkage (FBL) method in modern Chinese cotton cultivars or germplasm lines [5], [7]–[8], [10]–[11], [47]–[50], we found 28 consistent QTLs (28/59, 47.5%) between these four base cultivars and modern Chinese cotton cultivars (**Table 3**). This result indicates that these are stably transferred or inherited QTL which can be further used in marker-assisted selection (MAS) breeding to improve cotton yield and fiber quality in future.

**Table 3:** QTLs detected consistently between association- and FBL-mapping results.

Traits^a^	Association-mapped QTL			FBL mapped QTL	
	Marker loci	Chromosome	Position	QTL tagged in core cultivars	QTL tagged in modern cultivars
PB	NAU980	A11(Chr.11)	13.771	*qPB-A11-1*	
	dPL0308	A13(Chr.13)	82.479	*qPB-A13-1*	
	BNL3590	D3(Chr.17)	33.653	*qPB-D3-1*	
	NAU1322	D8(Chr.24)	88.411		qFN-D8-1 [48]
	JESPR092	D12(Chr.26)	87.602		qFN-D12-1 [48]
NB	NAU2679	A6(Chr.6)	79.793	*qNB-A6-1*	
	NAU5166	A10(Chr.10)	102.069	*qNB-A10-1*	
	NAU478	D8(Chr.24)	66.832		qBN-D8-1 [8]
					qBN-D8-1 [48]
	JESPR135	D11(Chr.21)	53.009	*qNB-D11-1*	
BW	NAU2272	D2(Chr.14)	57.155	*qBW-D2-1*	
	NAU2439	D8(Chr.24)	70.586		qBS-D8-1 [8]
	NAU3084	D12(Chr.26)	0		qBW-D12-1 [11]
LP	NAU3385	A1(Chr.1)	60.983	qLP-A1-1*	qLP-A1-1 [48]
	JESPR101	A2(Chr.2)	63.268	*qLP-A2-1*	
	NAU5166	A10(Chr.10)	102.069		qLP-A10-1 [5]
	NAU4024	D2(Chr.14)	42.074	qLP-D2-1*	qLP-D2-1 [8]
					qLP-D2-1 [5]
					qLP-D2-1 [48]
	NAU3700	D3(Chr.17)	59.833	*qLP-D3-1*	
	BNL448	D4(Chr.22)	25.671		qLP-D4-1 [8]
					qLP-D4-1 [11]
	NAU1042	D5(Chr.19)	132.967	*qLP-D5-1*	
	BNL3103	D6(Chr.25)	39.318		qLP-D6-1 [48]
	NAU4956	D7(Chr.16)	79.014		qLP-D7-1 [48]
	BNL252	D8(Chr.24)	98.304	*qLP-D8-1*	
	NAU3414	D9(Chr.23)	135.986		qLP-D9-1 [48]
	NAU3961	D12(Chr.26)	71.035		qLP-D12-1 [11]
	NAU2697	D13(Chr.18)	33.326	*qLP-D13-1*	
LI	NAU3385	A1(Chr.1)	60.983	*qLI-A1-1*	
	NAU1167	A3(Chr.3)	100.852	*qLI-A3-1*	
	NAU3269	A5(Chr.5)	177.423		qLI-A5-1 [11]
	NAU2165	D1(Chr.15)	62.181	*qLI-D1-1*	
	NAU2761	D3(Chr.17)	71.367	*qLI-D3-1*	
	JESPR220	D4(Chr.22)	105.942		qLI-D4-1 [5]
	NAU3092	D5(Chr.19)	180.734	*qLI-D5-1*	
	NAU2169	D8(Chr.24)	77.129		qLY-D8-1 [8]
SI	NAU2761	D3(Chr.17)	71.367		qSI-D3-1 [5]
	NAU1042	D5(Chr.19)	132.967	*qSI-D5-1*	
	NAU478	D8(Chr.24)	66.832	*qSI-D8-1*	
	NAU2439	D8(Chr.24)	70.586		qSI-D8-1 [8]
					qSI-D8-1 [11]
					qSI-D8-1 [48]
	JESPR135	D11(Chr.21)	53.009	*qSI-D11-1*	
FL	NAU2277	A2(Chr.2)	3.052	*qFL-A2-1*	
	NAU3273	A5(Chr.5)	37.291		qFL-A5-1 [8]
	NAU1048	A7(Chr.7)	53.906		qFL-7-1 [7]
					FL [47]
	NAU980	A11(Chr.11)	13.771		qFL-A11-1 [5]
	NAU3639	D3(Chr.17)	58.883		qFL-D3-1 [49]
	JESPR220	D4(Chr.22)	105.942		qFL-D4-1 [5]
	NAU3917	D10(Chr.20)	30.981	*qFL-D10-1*	
FS	cgr5565	A10(Chr.10)	113.977		qFS-A10-1 [5]
	NAU980	A11(Chr.11)	13.771		qFS-A11-1 [5]
	NAU3390	A11(Chr.11)	136.999		qFS-A11-1 [10]
	cgr5510	D5(Chr.19)	146.873	*qFS-D5-1*	
FM	NAU3061	A9(Chr.9)	112.15		FF1 [47]
	JESPR300	A12(Chr.12)	146.228		qFM-A12-1 [5]
	BNL3590	D3(Chr.17)	33.653		qFM-D3-1 [49]
	NAU2931	D7(Chr.16)	82.041		qFM-16-1b [7]
	NAU3053	D7(Chr.16)	98.316		qFM-D7-1 [49]
	NAU2169	D8(Chr.24)	77.129		qFMIC-D8-1 [8]
					FM2 [50]
	BNL946	D10(Chr.20)	58.164	qFM-D10-1*	FF2 [47]
	NAU3493	D11(Chr.21)	121.916		qFM-D11-1 [49]
	JESPR153	D13(Chr.18)	54.995		qFM-D13-1 [49]
					FM1 [50]
FU	NAU4946	A6(Chr.6)	90.228		qFU-A6-1 [5]
	NAU980	A11(Chr.11)	13.771		qFU-A11-1 [49]
	NAU3053	D7(Chr.16)	98.316		qFU-D7-1 [49]
FE	NAU3273	A5(Chr.5)	37.291		qFE-5-1a [7]
	NAU980	A11(Chr.11)	13.771		qFE-A11-1 [49]
	BNL1681	A11(Chr.11)	58.725		qFE-A11-1 [8]
	NAU4024	D2(Chr.14)	42.074	qFE-D2-1*	qFE-D2-1 [5]
					FE [50]
	cgr5510	D5(Chr.19)	146.873	qFE-D5-1*	qFE-D5-1 [5]
Total	74			35	55

a See Table 2 for abbreviations, and *QTL detected consistent.

### Population-based Association QTL Mapping for Yield and Fiber Qualities

LD is the basis of association mapping. The analysis of genome-wide LD between SSR loci provides markers for the status of LD in the cotton genome. In this study, the proportion of locus pairs supported by significant probability (P<0.01) was low and accounted for only 2.93% (624/21321), indicating that the level of LD in the cotton genome was low. We also determined the structure of haplotypic LD since a strong block-like LD structure simplifies LD mapping of complex traits. Triangle plots for pairwise LD between SSR markers demonstrated significant LD blocks in the genome-wide LD analysis. The decay rate of r^2^ values was very fast, the maximum distance of LD decay of cotton cultivars in this study was approximately 13–14 cM (Figure S1). The results of STRUCTURE showed that the Chinese Upland cotton cultivars (**Table 1**) could be best divided into four subgroups (Figure S2).

Performance of association mapping of SSR loci with 12 agronomic and fiber quality traits from the seven environments (06JS, 07JS, 08JS, 07XJ, 08XJ, 08HN and 08SD), resulted in detection of 180 loci that significantly associated with the traits (P<0.05) within more than one environment (Table S2). Out of these 121 SSRs, two SSRs (NAU980 on chro.A11 and JESPR220 on chro.D4) were associated with six traits, two SSRs (NAU3053 on chro.D7 and TMH05 on chro.D11) with five traits, 11 SSRs (BNL3280, BNL3590, JESPR101, JESPR135, JESPR232, NAU3084, NAU3206, NAU3917, NAU422, NAU4956 and NAU5166) with four traits, 20 SSRs with three traits and 29 SSRs with two traits, and the remaining 57 SSRs each with one trait (Table S3).

QTLs for yield and fiber qualities detected in 4WC, LD association and FBL QTL mapping in modern Chinese cotton cultivars are summarized in **Table 3**. There were 66 population-based QTL associations for 12 yield and fiber quality traits which we detected either in 4WC or FBL mapping in modern Chinese cotton cultivars. By comparing 4WC mapping and LD mapping, we found that there were 28 consistent QTLs (28/180, 15.56%) between them. Furthermore, the 44 consistent QTLs (44/180, 24.44%) which were mapped in modern Chinese cotton cultivars using conventional FBL and LD mapping (**Table 3**), revealed that these are stably inherited QTLs which can be used in MAS breeding. We believe that the more the cotton cultivars are used to tag QTL and the more the consistent QTLs will be detected.

### Mining of Elite QTL Alleles to Improve Yield and Fiber Qualities in Cotton

Among the 402 amplified SSRs, 207 appeared polymorphic and produced a total of 541 alleles. The average number of alleles per locus was 2.61, ranging from 2 to 7. More than half of the primers amplifying polymorphic alleles (120 SSR primers) generated two alleles. The large range and the low mean value indicated that the variation of cotton cultivars was rich at the genome level, but that the genetic basis of variation in Upland cotton was limited.

Phenotypic effects of some elite QTL alleles significantly associated with agronomic and fiber quality traits and their typical characteristics are shown in Table S3. Each QTL allele had positive and/or negative alleles to some extents. Among the alleles associated with LP, qNAU3398-3 in Simian 4 had the most positive phenotypic effect and was able to increase LP by 8.26%, whereas NAU5166-3 in Shanmian1 had the most negative phenotypic effect (−11.49%). Among the alleles associated with PH, qNAU5091-2 had the most positive phenotypic effect (5.10 cm), whereas qJESPR232-2 and qJESPR227-2 had the most negative phenotypic effect (−18.3 cm). Among the alleles of loci associated with FS, qNAU2156-2 in CRI4133 had the most positive phenotypic effect and increased fiber strength to 1.80 cN/tex while qNAU2156-3 in 52–128 had the most negative phenotypic effect (−0.94 cN/tex).

### Transmission and Variations of QTL Alleles for Yield and Fiber Qualities among Chinese Cotton Cultivars

The transmission and variation of elite QTL alleles for each trait in the three breeding periods are summarized in **Table 4**. From this table it is obvious which QTL allele was passed down from the four core cultivars, which ones detected to exist in the four core cultivars and were not selected by breeders to develop modern Chinese cotton cultivars, and which ones were new and/or unreported QTL alleles associated with agronomic and fiber quality traits. It enabled us to classify QTL alleles detected in the present study into three types and this is illustrated using lint percentage as an example(**Table 4**). The first type of QTL alleles, such as qNAU3917-1 and qBNL3103-1, can be detected in all four core cultivars and were transferred into most cultivars in the two breeding periods. These QTL alleles should be regarded as base genetic constitution for lint development. The second type, such as qNAU1302-1 and qNAU3700-1, were detected in three core cultivars and transferred into some of the cultivars during the two breeding periods. The third type, such as qNAU5166-2 and qNAU3398-3, which can greatly increase lint percentage by 6.48% and 8.26%, respectively, were neither found in the four core cultivars nor in most Chinese cultivars. These QTL alleles may have been introduced from other sources, perhaps by genetic recombination, and have a great potential in increasing lint percentage and lint yield in MAS breeding.

**Table 4:** Transmission and variations of QTL alleles for yield and fiber qualities among Chinese cotton cultivars.

Traits^a^	Markers	a_i_	Typical genotype	Period		
				I(1^b^-5)	II(6–35)	III(36–81)
PB	NAU3419-2	0.21	Jimian12	2	6,7,8,10,17–19,21,25,28,29,31,32,34	37,39,41,43–45,48,51,54,57,61,62,69,71,74,76
	BNL3792-2	0.20	Xuzhou1818	2	7,17,19,31,32,33	52,53,54,58,69
	BNL3792-3	2.04	Shanmian1	No	21	No
	NAU4956-2	2.04	Shanmian1	No	21	No
	NAU4956-3	0.22	Xuzhou1818	No	19	No
	NAU3917-2	2.04	Shanmian1	No	21	No
	TMH05-1	0.35	Xuzhou1818	1	6,8,11,18,19,21,31	39,41,62
NB	JESPR101-1	0.72	Shanmian4	1	Except8,16,17,19,21,24,25,27,29–33	Except38–40,44,48,50,52,54,57,63–65,68–71,76–78,80
	BNL3590-1	0.55	Sumian6	1,3,5	Except8,16,17,19,21,24,27,29–31	Except38,39,44,50,57,78
	NAU3700-1	0.30	Sumian6	1,3,5	Except 8,12,16,17,19–21,29,31	Except 56,78
	NAU3084-1	0.24	Sumian16	1,2,3,5	Except 7,21,24,31	Except 39,59,69
BW(g)	NAU3269-2	0.17	CRI34	1,3,5	6–8,11,17,19,22–24,31	Except 37,38,40,43,45–47,50–52,54,56,57,59,63–72,74–76
	BNL3280-3	0.11	Keyi2	5	6,12,17	55
LP(%)	NAU5166-2	6.48	Simian3	No	No	69
	NAU3398-2	1.88	Ejing1	No	26,27,30	43,46,60,72,79,80
	NAU3398-3	8.26	Simian4	No	No	70
	NAU3700-1	0.57	CRI16	1,3,5	Except 8,12,16,17,19,21,29,31	Except 56,78
	BNL448-2	5.17	8891	No	No	60,70,72,79,80
	BNL3103-1	0.19	CRI16	All	Except 20,21	All
	NAU1302-1	0.49	CRI16	2,3,5	Except 6,12,14,21,27	Except 40,48,50,61,72,74,78
	NAU3917-1	0.15	CRI16	All	Except 21	All
	NAU2697-1	0.71	CRI16	3,4,5	Except6,8,10,11,19,21,25,29,32,34,35	Except 36,39,50,61,62,74–76
LI(g)	NAU1254-2	0.31	Lumian6	3,4	8,10,11,16,18,22,27,30,32,34,35	Except 37–40,45,47,50–54,56,59,73,77–81
	NAU3398-3	0.80	Simian4	No	No	70
	BNL448-2	0.15	8891	No	No	60,70,72,79,80
	NAU1302-1	0.05	DPL16	2,3,5	Except 6,12,14,21,27	Except 40,48,50,61,72,74,78
SI(g)	JESPR101-2	0.65	57-681	2	8,16,17,19,21,29,31	44,78
	JESPR101-4	0.83	Shan1155	No	25,30	52,57
	JESPR101-5	2.44	Yumian5	4	No	39
	NAU1151-2	0.66	CRI19	1,2	6,8,11,18,22,26,30,31,34,35	39,41–44,50,52,56,58,62
	JESPR274-1	0.18	TM-1	1,3,4,5	Except 7,15,16,19–21,24,31,32,35	Except36,38,39,43,44,46–48,54–57,62,65,74–79,81
	NAU4921-2	1.36	Jingsimian	No	8,17,24	39,42
	JESPR135-2	1.83	Jingsimian	No	8,21	39,57,78
FL(mm)	NAU1048-1	0.20	Sumian6	1	6–8,13,17–19,24,25,28,35	39,47,48,52,55,57–59,62,64,66,69,71,81
	NAU4921-2	0.62	DPL16	No	8,17,24	39,42
FS(cN/tex)	NAU422-2	0.07	Lumian4	1,2,3,4	Except 10,14,27	Except 41–43,57,61,72,75,80
	NAU2156-2	1.80	CRI4133	No	No	51,56,58
	cgr5510-2	0.12	Shan6192	2,3,4,5	Except 8,12,16,20,21,35	Except 38,43,59,61,65,73,75,76
FM	NAU1162-1	0.07	Sumian9	1,2,5	7,9,13–17,19,20,22–25,29–31	Except 37–41,43,48,49,54,60–63,71–73,75,77
	NAU3053-2	0.24	XiangSC-24	No	12	60,72,79
	NAU3053-3	0.55	Lumian5	No	33	44
FU(%)	NAU3061-1	0.09	CRI3	All	Except 9,17,26,34,35	Except 38,41–43,49,54,59,62,65,67,68,74
	NAU980-3	0.33	8891	No	16,24	60,72,80
	cgr5510-2	0.08	CRI3	2,3,4,5	Except 8,12,16,20,21,35	Except 38,43,59,61,65,73,75,76
FE(%)	NAU2156-3	0.41	52–128	No	10	No
	BNL1681-1	0.06	Lumian5	3,4,5	Except 6,11,29,35	Except 36,38,39,45,48–51,57,58,62,76,79,81

a See Table 2 for abbreviations.

b The numbers represent cultivars listed in Table 1.

## Discussion

In the present study, we successfully identified 180 QTL using 121 SSR markers and these were significantly associated with 12 agronomic and fiber quality traits. Among them, we identified 66 QTL *via* LD mapping for 12 yield and fiber quality traits which we detected either by 4WC or FBL mapping in some modern Chinese cotton cultivars. We found that there were 28 consistent QTLs between our 4WC and LD association mapping, and 44 consistent QTLs mapped in modern Chinese cotton cultivars using conventional FBL and LD mapping methods. Comparison of 4WC, LD association and FBL QTL mapping suggested that some of these QTLs were transmitted and/or kept in conventional breeding selection from the four introduced core cultivars and may be very important in cotton agronomic and fiber quality development. Our results revealed that association mapping based on LD using diverse sets of cultivated cotton germplasm is a useful tool in detecting QTLs efficiently.

### Association Mapping Based on LD is an Alternative Powerful Tool to Exploit the Natural Genetic Diversity in Cotton

The application of LD-based association mapping is an alternative powerful molecular tool to exploit the natural genetic diversity conserved within crop germplasm collections. The resolution of association mapping depends on the extent and distribution of LD across the genome within a given population [51]. The extent of LD has been scaled and association mapping has been successfully used in many plant species [21]. In sugar beet (*Beta vulgaris* L.), genome-wide LD extended up to 3 cM [52], but in some Arabidopsis populations, LD exceeded 50 cM [53]. Genome-wide LD decay as a function of genetic distance is very common for distances <10 cM [54] in barley (*Hordeum vulgare* L.), and very different in maize (*Zea mays* L.), in which LD diminished after 2000 bps [51].

Though association mapping based on LD was successfully used in some crops, it is important to consider the influence of mixed population structure and relationship of individuals in association mapping [42], [44], [55]. Many crops have a long and complex history of domestication and breeding, and complex population structures may confound association mapping [56]–[57].

Overall, the small extent of LD in the cotton genome illustrates the significant potential for LD-based association mapping for agronomic and fiber quality traits in cotton with a relatively large number of various sorts of markers. However, the limited polymorphism between Upland cotton cultivars may reduce the mapping resolution, particularly in breeding germplasm. As cross-pollination is common in cotton, the LD level in cotton genomes was low and only 2.95% of locus pairs were significant. LD decay was measured at 13–14 cM in cotton. Considering the tetraploid cotton genome with a total recombination length of about 5,200 cM and an average 400 kb per cM [58], the LD block sizes are still small to conduct association mapping of complex traits which would require nearly 1000 polymorphic markers. It is difficult to reach such a high density using only SSR markers, highlighting the need for new molecular markers. As next generation sequencing techniques develop, any progress to sequence tetraploid cotton will advance association mapping of complex traits based on single nucleotide polymorphisms.

### Potential Usages of QTL Alleles Identified in Genomics-assisted Cotton Breeding in Future

Association mapping based on LD using a GLM approach with 81 Upland cotton cultivars laid the foundation for a potential genomics-assisted breeding program in cotton. We analyzed SSR markers significantly associated with genotypes and phenotypes of cultivars in the average environment of every trait, and detected a number of elite alleles associated with 12 agronomic and fiber quality traits in Upland cotton. These will be useful for MAS breeding program to develop cultivars with high yield and superior fiber qualities. We suggest that a genomics-assisted ranking system for QTL alleles should be developed based on LD association mapping. First of all, great attention should be paid to those QTL alleles that are not found in the four core cultivars and in most other Chinese cultivars. They may have been introduced from other sources *via* genetic recombination and may hold great potential in increasing lint yield and fiber qualities. For example, qNAU5166-2 and qNAU3398-3 increased lint percentage by 6.48% and 8.26%, respectively. The more cotton germplasm lines are surveyed, the more elite QTL alleles may be mined.

Secondly, using MAS breeding, it would be prudent to select those QTL alleles which can be detected in all four core cultivars and most other Chinese cultivars since they may represent a basic genetic requirement. Examples are qNAU3917-1 and qBNL3103-1. In addition, QTL alleles which were detected in the core cultivars, but not in most of Chinese cultivars (such as qNAU1302-1 and qNAU3700-1) may represent desirable traits.

Thirdly, a genomics-assisted breeding program to pyramid QTL alleles could be developed on the basis of LD association mapping. For example, in our study qBNL3792-3 was associated with an increase in the number of cotton fruit branches, qNAU5166-2 was associated with enhanced lint percentage, qNAU4921-2 contributed to increased fiber length, and qNAU2156-2 associated with fiber strength. In view of specific links between phenotype and genotype, when selecting mating parents one should consider phenotype and genotype to achieve maximum complementary between materials. To improve fiber quality, for example, one should hybridize simultaneously the material with alleles qNAU4921-2 and qNAU1048-1, which can enhance fiber length efficiently, and another allele qNAU2156-2 which can increase fiber strength. It will then be possible to select cultivars with superior fiber qualities from their offspring through MAS programs.

## Supporting Information

Figure S1 LD decays within a distance.
(DOC)

Figure S2 The summary plots of Q-matrix estimates for the variety accessions.
(DOC)

Table S1 Summary of the location and the effects of QTL using interval mapping method in 4WC.
(DOC)

Table S2 Association loci for fiber qualities and yield components.
(DOC)

Table S3 Phenotypic effect of QTL alleles significantly associated with traits.
(DOC)
