# Supplementary material for: Variations and Transmission of QTL Alleles for Yield and Fiber Qualities in Upland Cotton Cultivars Developed in China
Source: PLoS One. 2013 Feb 27;8(2):e57220. doi: 10.1371/journal.pone.0057220 (PMC3584144; doi:10.1371/journal.pone.0057220)
Supplement: Figure S1 — LD decays within a distance. (DOC) [file pone.0057220.s001.doc]

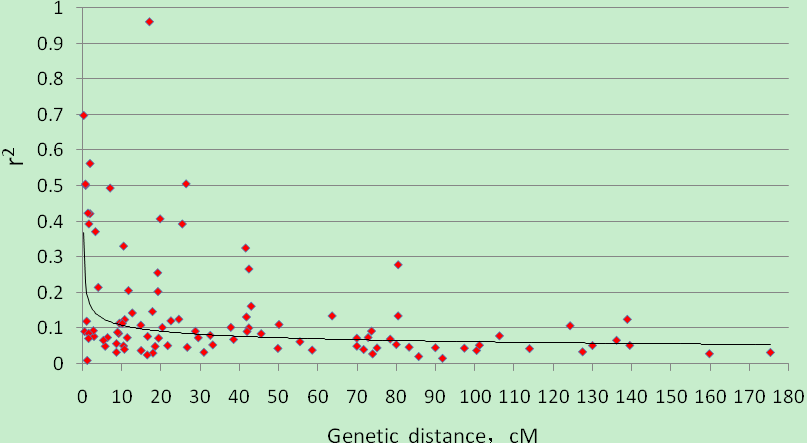


**Figure S1** LD decays within a distance.

Inner fitted trend line is a non-linear logarithmic regression curve of r^2^ on genetic distance. LD-decay is considered below r^2^ = 0.1 threshold based on trend line (Witt and Buckler 2003)
