# Supplementary figures and images for: Variations and Transmission of QTL Alleles for Yield and Fiber Qualities in Upland Cotton Cultivars Developed in China
Source: PLoS One. 2013 Feb 27;8(2):e57220. doi: 10.1371/journal.pone.0057220 (PMC3584144; doi:10.1371/journal.pone.0057220)

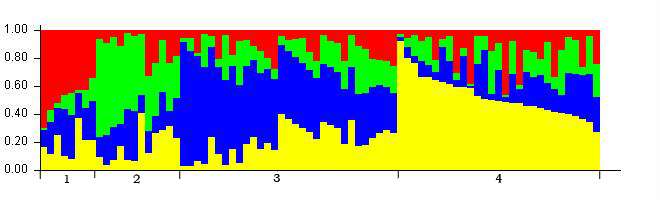


**Figure S2** The summary plots of Q-matrix estimates for the variety accessions

Supplement: Figure S2 — The summary plots of Q-matrix estimates for the variety accessions. (DOC) [file pone.0057220.s002.doc]
