# Supplementary material for: Variations and Transmission of QTL Alleles for Yield and Fiber Qualities in Upland Cotton Cultivars Developed in China
Source: PLoS One. 2013 Feb 27;8(2):e57220. doi: 10.1371/journal.pone.0057220 (PMC3584144; doi:10.1371/journal.pone.0057220)
Supplement: Table S1 — Summary of the location and the effects of QTL using interval mapping method in 4WC. (DOC) [file pone.0057220.s003.doc]

Table S1 Summary of the location and the effects of QTL using interval mapping method in 4WC

| Trait ^a^ | QTL | Generation | Position | Nearest marker | LOD | LOD^b^ | LOD^c^ | G_13_^d^ | G_14_^d^ | G_23_^d^ | G_24_^d^ | PVE(%)^e^ | a_1_^f^ | a_2_^g^ | d^h^ |
| --- | --- | --- | --- | --- | --- | --- | --- | --- | --- | --- | --- | --- | --- | --- | --- |
|
| PH | *qPH-A1-1* | F_2:3_ | 44.25 | NAU3135 | 3.64 | 1.5 | 3.9 | 101.34 | 101.34 | 97.29 | 97.29 | 10.50 | 2.02 | 0.00 | 0.00 |
|  | *qPH-A7-1* | F_2_、F_2:3_ | 19.79 | NAU1048 | 4.32* | 1.2 | 4.3 | 85.21 | 85.21 | 91.57 | 91.57 | 2.90 | -3.18 | 0.00 | 0.00 |
|  | *qPH-A9-1* | F_2_ | 0.00 | JESPR274 | 1.41 | 1 | 4.3 | 85.27 | 91.92 | 85.27 | 91.92 | 3.10 | 0.00 | -3.33 | 0.00 |
|  | *qPH-A11-1* | F_2_ | 0.00 | NAU3074 | 2.88 | 2.7 | 4.3 | 66.02 | 101.44 | 100.63 | 72.27 | 72.50 | -1.36 | -1.76 | -15.95 |
|  | *qPH-A13-1* | F_2_ | 23.39 | NAU1141 | 3.71 | 2.8 | 4.3 | 69.70 | 102.12 | 100.19 | 70.80 | 67.80 | 0.21 | -0.76 | -15.45 |
|  | *qPH-D1-1* | F_2_ | 50.61 | NAU2165 | 4.83* | 3 | 4.3 | 103.27 | 98.32 | 68.85 | 67.03 | 73.90 | 16.43 | 1.69 | 0.78 |
|  | *qPH-D6-1* | F_2_ | 90.17 | BNL3806 | 3.45 | 2.9 | 4.3 | 87.01 | 102.89 | 100.25 | 66.29 | 60.30 | 5.84 | 4.52 | -12.46 |
|  | *qPH-D7-1* | F_2_ | 41.46 | NAU2862 | 4.51* | 2.6 | 4.3 | 102.82 | 99.31 | 72.09 | 66.81 | 70.80 | 15.81 | 2.20 | -0.45 |
|  | *qPH-D7-2* | F_2_ | 9.59 | cgr5656 | 3.45 | 1.8 | 4.3 | 81.04 | 96.49 | 92.62 | 72.32 | 21.40 | 3.15 | 1.21 | -8.94 |
|  | *qPH-D9-1* | F_2_ | 20.77 | dPL0783 | 1.47 | 1.2 | 4.3 | 85.06 | 85.06 | 91.88 | 91.88 | 3.30 | -3.41 | 0.00 | 0.00 |
| NB | *qNB-A4-1* | F_2:3_ | 7.00 | NAU5426 | 2.05 | 1.1 | 3.9 | 11.36 | 11.36 | 12.52 | 12.52 | 5.20 | -0.58 | 0.00 | 0.00 |
|  | *qNB-A6-1* | F_2_ | 22.20 | NAU5433 | 3.70 | 2.2 | 4 | 14.84 | 16.24 | 19.29 | 16.07 | 10.50 | -1.07 | 0.45 | -1.15 |
|  | *qNB-A7-1* | F_2:3_ | 19.79 | NAU1048 | 1.44 | 1.3 | 3.9 | 12.35 | 12.35 | 11.42 | 11.42 | 3.30 | 0.46 | 0.00 | 0.00 |
|  | *qNB-A9-1* | F_2_ | 0.00 | JESPR274 | 1.45 | 1.1 | 4 | 15.77 | 17.39 | 15.77 | 17.39 | 3.00 | 0.00 | -0.81 | 0.00 |
|  | *qNB-A10-1* | F_2_ | 48.65 | cgr5399 | 2.18 | 1.3 | 4 | 15.51 | 15.51 | 17.49 | 17.49 | 4.60 | -0.99 | 0.00 | 0.00 |
|  | *qNB-D5-1* | F_2_ | 30.82 | cgr5510 | 1.66 | 1.2 | 4 | 15.50 | 15.50 | 17.25 | 17.25 | 3.50 | -0.88 | 0.00 | 0.00 |
|  | *qNB-D7-1* | F_2_ | 1.00 | NAU2931 | 3.17 | 2.4 | 4 | 18.52 | 15.97 | 15.16 | 16.44 | 7.20 | 0.72 | 0.32 | 0.96 |
|  | *qNB-D7-2* | F_2_ | 9.59 | cgr5656 | 2.58 | 2.4 | 4 | 15.42 | 16.07 | 22.03 | 18.18 | 14.70 | -2.18 | 0.80 | -1.12 |
|  | *qNB-D9-1* | F_2_ | 17.09 | dPL0783 | 1.80 | 1.2 | 4 | 15.61 | 15.61 | 17.51 | 17.51 | 4.20 | -0.95 | 0.00 | 0.00 |
|  | *qNB-D11-1* | F_2_ | 39.13 | JESPR135 | 1.42 | 1.3 | 4 | 15.74 | 15.74 | 17.43 | 17.43 | 3.20 | -0.84 | 0.00 | 0.00 |
| PB | *qPB-A11-1* | F_2_ | 0.00 | NAU3074 | 4.98 | 4.3 | 5.2 | 12.74 | 18.62 | 18.86 | 13.10 | 61.30 | -0.15 | -0.03 | -2.91 |
|  | *qPB-A13-1* | F_2_ | 12.62 | dPL0308 | 2.52 | 2.2 | 5.2 | 14.58 | 17.68 | 17.05 | 15.38 | 11.20 | -0.04 | -0.36 | -1.19 |
|  | *qPB-D2-1* | F_2_ | 63.21 | NAU4024 | 3.19 | 2.7 | 5.2 | 17.71 | 12.71 | 17.75 | 15.68 | 28.50 | -0.75 | 1.77 | 0.73 |
|  | *qPB-D3-1* | F_2:3_ | 26.74 | JESPR101b | 2.14 | 2 | 4.9 | 16.12 | 15.08 | 15.16 | 15.25 | 4.90 | 0.20 | 0.23 | 0.28 |
|  | *qPB-D6-1* | F_2:3_ | 53.87 | dPL0124 | 3.14 | 2.7 | 4.9 | 16.00 | 15.70 | 15.17 | 14.67 | 7.50 | 0.46 | 0.20 | -0.05 |
|  | *qPB-D13-1* | F_2:3_ | 8.00 | NAU3232 | 1.75 | 1.6 | 4.9 | 15.80 | 15.80 | 14.94 | 14.94 | 5.80 | 0.43 | 0.00 | 0.00 |
|  | *qPB-D13-2* | F_2_ | 0.00 | NAU680a | 2.00 | 1.4 | 5.2 | 15.43 | 16.94 | 15.43 | 16.94 | 4.20 | 0.00 | -0.76 | 0.00 |
| BW | *qBW-D2-1* | F_2_ | 20.23 | NAU2272b | 4.25* | 2.7 | 3.8 | 5.93 | 4.57 | 5.62 | 5.35 | 13.10 | -0.12 | 0.41 | 0.28 |
|  | *qBW-D3-1* | F_2:3_ | 19.84 | NAU5386 | 1.53 | 1.2 | 3.8 | 5.75 | 5.75 | 5.59 | 5.59 | 3.50 | 0.08 | 0.00 | 0.00 |
|  | *qBW-D3-2* | F_2:3_ | 22.49 | NAU2761 | 2.27 | 2.2 | 3.8 | 5.62 | 5.91 | 5.47 | 5.71 | 13.50 | 0.09 | -0.13 | -0.02 |
|  | *qBW-D6-1* | F_2_ | 82.84 | NAU2063 | 3.31 | 2.7 | 3.8 | 5.92 | 5.28 | 5.97 | 5.16 | 13.60 | 0.02 | 0.36 | -0.04 |
| LP | *qLP-A1-1* | F_2:3_ | 0.00 | NAU2741 | 1.35 | 1.2 | 4.4 | 0.36 | 0.36 | 0.35 | 0.35 | 3.10 | 0.00 | 0.00 | 0.00 |
|  | *qLP-A2-1* | F_2_、F_2:3_ | 1.87 | BNL3590 | 4.90* | 1.5 | 4.4 | 0.36 | 0.36 | 0.36 | 0.35 | 10.70 | 0.00 | 0.00 | 0.00 |
|  | *qLP-A3-1* | F_2_ | 51.55 | BNL226 | 3.34 | 1.4 | 3.7 | 0.30 | 0.32 | 0.30 | 0.32 | 6.80 | 0.00 | -0.01 | 0.00 |
|  | *qLP-D1-1* | F_2:3_ | 23.72 | NAU3018 | 4.04 | 2.4 | 4.4 | 0.35 | 0.35 | 0.35 | 0.37 | 28.30 | 0.00 | -0.01 | 0.01 |
|  | *qLP-D2-1* | F_2:3_ | 4.00 | NAU5125 | 3.06 | 2.9 | 4.4 | 0.35 | 0.38 | 0.36 | 0.35 | 32.20 | 0.01 | -0.01 | -0.01 |
|  | *qLP-D3-1* | F_2_、F_2:3_ | 24.49 | NAU3700 | 5.18* | 2.2 | 4.4 | 0.37 | 0.35 | 0.36 | 0.35 | 12.10 | 0.00 | 0.00 | 0.00 |
|  | *qLP-D5-1* | F_2:3_ | 30.82 | cgr5510 | 1.86 | 1.2 | 4.4 | 0.36 | 0.36 | 0.35 | 0.35 | 4.30 | 0.00 | 0.00 | 0.00 |
|  | *qLP-D8-1* | F_2_ | 39.34 | BNL252 | 1.81 | 1.4 | 3.7 | 0.30 | 0.31 | 0.30 | 0.31 | 4.90 | 0.00 | -0.01 | 0.00 |
|  | *qLP-D13-1* | F_2:3_ | 0.00 | cgr5390 | 1.16 | 1.1 | 4.4 | 0.36 | 0.36 | 0.35 | 0.35 | 2.70 | 0.00 | 0.00 | 0.00 |
|  | *qLP-LG1-1* | F_2:3_ | 69.11 | cgr6806 | 1.79 | 1.5 | 4.4 | 0.36 | 0.36 | 0.35 | 0.35 | 6.50 | 0.00 | 0.00 | 0.00 |
|  | *qLP-LG2-1* | F_2_ | 11.09 | NAU7477 | 1.39 | 1.1 | 3.7 | 0.30 | 0.31 | 0.30 | 0.31 | 2.90 | 0.00 | 0.00 | 0.00 |
|  | *qLP-LG3-1* | F_2:3_ | 34.00 | NAU2932 | 1.16 | 1.1 | 4.4 | 0.35 | 0.36 | 0.35 | 0.36 | 2.90 | 0.00 | 0.00 | 0.00 |
| LI | *qLI-A1-1* | F_2:3_ | 0.00 | NAU2741 | 2.08 | 1.2 | 3.9 | 6.50 | 6.50 | 6.30 | 6.30 | 4.70 | 0.10 | 0.00 | 0.00 |
|  | *qLI-A3-1* | F_2_ | 46.80 | NAU5233 | 1.26 | 1.2 | 3.8 | 5.18 | 5.18 | 4.93 | 4.93 | 2.70 | 0.13 | 0.00 | 0.00 |
|  | *qLI-A4-1* | F_2_ | 0.00 | BNL530 | 2.32 | 2.1 | 3.8 | 4.76 | 5.09 | 5.20 | 5.09 | 4.90 | -0.11 | -0.05 | -0.11 |
|  | *qLI-A9-1* | F_2_ | 25.10 | NAU5198 | 1.27 | 1.2 | 3.8 | 4.89 | 4.89 | 5.14 | 5.14 | 2.60 | -0.12 | 0.00 | 0.00 |
|  | *qLI-D1-1* | F_2:3_ | 24.72 | dPL0542 | 3.68 | 2.3 | 3.9 | 6.29 | 6.38 | 6.26 | 6.77 | 16.70 | -0.09 | -0.15 | 0.11 |
|  | *qLI-D2-1* | F_2:3_ | 10.00 | NAU2272b | 3.61 | 2.7 | 3.9 | 6.45 | 6.97 | 6.38 | 6.07 | 25.80 | 0.24 | -0.05 | -0.21 |
|  | *qLI-D3-1* | F_2:3_ | 22.49 | NAU2761 | 4.21* | 2.1 | 3.9 | 6.67 | 6.46 | 6.31 | 6.27 | 10.20 | 0.14 | 0.06 | 0.04 |
|  | *qLI-D5-1* | F_2_ | 5.48 | NAU3609 | 1.24 | 1 | 3.8 | 5.13 | 5.13 | 4.89 | 4.89 | 2.60 | 0.12 | 0.00 | 0.00 |
|  | *qLI-LG4-1* | F_2_ | 28.05 | cgr5108 | 2.11 | 2 | 3.8 | 4.61 | 5.28 | 5.03 | 5.21 | 12.30 | -0.09 | -0.21 | -0.12 |
| SI | *qSI-A9-1* | F_2_ | 21.00 | NAU5198 | 2.67 | 1 | 4.8 | 10.87 | 10.87 | 11.59 | 11.59 | 6.50 | -0.36 | 0.00 | 0.00 |
|  | *qSI-A12-1* | F_2:3_ | 49.81 | cgr5452 | 1.73 | 1.3 | 3.7 | 11.35 | 11.35 | 11.66 | 11.66 | 4.10 | -0.16 | 0.00 | 0.00 |
|  | *qSI-D5-1* | F_2:3_ | 0.00 | NAU3217 | 2.77 | 1.2 | 3.7 | 11.37 | 11.37 | 11.73 | 11.73 | 6.20 | -0.18 | 0.00 | 0.00 |
|  | *qSI-D7-1* | F_2:3_ | 0.00 | NAU2627 | 1.63 | 1 | 3.7 | 11.43 | 11.43 | 11.71 | 11.71 | 3.90 | -0.14 | 0.00 | 0.00 |
|  | *qSI-D8-1* | F_2:3_ | 29.96 | NAU3786a | 1.56 | 1.3 | 3.7 | 11.70 | 11.41 | 11.70 | 11.41 | 4.20 | 0.00 | 0.15 | 0.00 |
|  | *qSI-D11-1* | F_2_ | 4.00 | NAU2141 | 2.92 | 1.4 | 4.8 | 10.92 | 10.92 | 11.69 | 11.69 | 7.40 | -0.38 | 0.00 | 0.00 |
|  | *qSI-LG3-1* | F_2_ | 36.85 | NAU2932 | 1.73 | 1.2 | 4.8 | 11.51 | 10.97 | 11.51 | 10.97 | 3.60 | 0.00 | 0.27 | 0.00 |
| FL | *qFL-A2-1* | F_2_ | 13.91 | NAU2277 | 2.57 | 2.3 | 3.9 | 28.03 | 26.80 | 28.32 | 26.91 | 31.60 | -0.10 | 0.66 | -0.05 |
|  | *qFL-A10-1* | F_2:3_ | 45.81 | cgr5565 | 2.44 | 1.3 | 4 | 28.14 | 28.14 | 28.57 | 28.57 | 5.80 | -0.21 | 0.00 | 0.00 |
|  | *qFL-A10-2* | F_2:3_ | 7.53 | cer0063 | 1.39 | 1.1 | 4 | 28.19 | 28.19 | 28.51 | 28.51 | 3.20 | -0.16 | 0.00 | 0.00 |
|  | *qFL-D2-1* | F_2:3_ | 20.78 | NAU5104 | 1.33 | 1.2 | 4 | 28.51 | 28.51 | 28.20 | 28.20 | 3.10 | 0.15 | 0.00 | 0.00 |
|  | *qFL-D10-1* | F_2:3_ | 13.00 | cgr6022 | 1.98 | 1.3 | 4 | 28.58 | 28.13 | 28.58 | 28.13 | 6.50 | 0.00 | 0.22 | 0.00 |
|  | *qFL-D12-1* | F_2:3_ | 0.00 | dPL0404 | 1.63 | 1.3 | 4 | 28.18 | 28.52 | 28.18 | 28.52 | 3.80 | 0.00 | -0.17 | 0.00 |
|  | *qFL-D13-1* | F_2:3_ | 26.12 | BNL3280 | 1.61 | 1.6 | 4 | 28.53 | 28.53 | 28.13 | 28.13 | 5.40 | 0.20 | 0.00 | 0.00 |
| FS | *qFS-A12-1* | F_2:3_ | 25.95 | NAU4926 | 3.62 | 2.3 | 4.1 | 30.36 | 29.25 | 30.59 | 30.38 | 11.10 | -0.34 | 0.33 | 0.23 |
|  | *qFS-D5-1* | F_2:3_ | 4.00 | dPL0056 | 2.08 | 1.3 | 4.1 | 29.78 | 29.78 | 30.51 | 30.51 | 6.00 | -0.37 | 0.00 | 0.00 |
| FM | *qFM-D10-1* | F_2:3_ | 17.00 | BNL119 | 1.23 | 1.1 | 3.7 | 4.98 | 4.98 | 4.85 | 4.85 | 3.60 | 0.07 | 0.00 | 0.00 |
|  | *qFM-D12-1* | F_2_ | 13.00 | dPL0404 | 2.04 | 1.1 | 4.2 | 4.38 | 4.09 | 4.38 | 4.09 | 7.90 | 0.00 | 0.14 | 0.00 |
| FE | *qFE-A2-1* | F_2:3_ | 0.00 | NAU3419 | 3.14 | 2.1 | 3.7 | 5.31 | 6.12 | 5.70 | 5.70 | 28.90 | 0.01 | -0.20 | -0.20 |
|  | *qFE-A3-1* | F_2:3_ | 1.00 | dPL0609 | 2.66 | 1.4 | 3.7 | 5.57 | 5.84 | 5.57 | 5.84 | 6.50 | 0.00 | -0.14 | 0.00 |
|  | *qFE-A4-1* | F_2_ | 10.04 | BNL3994 | 2.40 | 2.2 | 3.9 | 6.57 | 5.80 | 5.96 | 5.84 | 7.90 | 0.14 | 0.22 | 0.16 |
|  | *qFE-A12-1* | F_2:3_ | 35.43 | cgr5193 | 3.22 | 2.3 | 3.7 | 5.56 | 5.99 | 5.62 | 5.68 | 9.40 | 0.06 | -0.12 | -0.09 |
|  | *qFE-A12-2* | F_2_ | 0.00 | NAU3778 | 1.08 | 1 | 3.9 | 6.24 | 5.88 | 6.24 | 5.88 | 2.60 | 0.00 | 0.18 | 0.00 |
|  | *qFE-D2-1* | F_2_ | 16.00 | NAU2272b | 3.97* | 2.2 | 3.9 | 5.93 | 9.27 | 5.67 | 6.04 | 53.30 | 0.87 | -0.93 | -0.74 |
|  | *qFE-D5-1* | F_2:3_ | 7.00 | NAU3217 | 2.50 | 1.3 | 3.7 | 5.86 | 5.86 | 5.56 | 5.56 | 7.70 | 0.15 | 0.00 | 0.00 |
| FU | *qFU-A2-1* | F_2_ | 1.24 | JESPR101a | 2.36 | 1.5 | 4 | 81.97 | 81.68 | 81.68 | 82.63 | 5.60 | -0.17 | -0.17 | 0.31 |
|  | *qFU-A12-1* | F_2:3_ | 3.00 | NAU3006 | 2.69 | 2.3 | 3.8 | 83.75 | 82.98 | 83.56 | 83.94 | 12.80 | -0.20 | 0.10 | 0.29 |
|  | *qFU-D1-1* | F_2:3_ | 0.00 | NAU1600 | 3.36 | 2.3 | 3.8 | 83.51 | 83.24 | 83.14 | 84.42 | 19.20 | -0.21 | -0.25 | 0.39 |
|  | *qFU-D2-1* | F_2:3_ | 10.00 | NAU1490 | 1.57 | 1.2 | 3.8 | 83.74 | 83.74 | 83.31 | 83.31 | 4.40 | 0.21 | 0.00 | 0.00 |
|  | *qFU-D7-1* | F_2_ | 0.00 | NAU2627 | 1.27 | 1 | 4 | 81.70 | 81.70 | 82.31 | 82.31 | 3.20 | -0.31 | 0.00 | 0.00 |
|  | *qFU-D12-1* | F_2_ | 28.86 | dPL0917 | 1.31 | 1.1 | 4 | 82.33 | 81.72 | 82.33 | 81.72 | 3.10 | 0.00 | 0.30 | 0.00 |

Plant height (PH, cm), number of fruit branches per plant (PB), number of bolls per plant (NB), boll weight (BW, g), lint percentage (LP), lint index (LI) and seed index (SI). Lint yield (LY) was determined by multiplying lint percentage with total seed cotton weight. The following fiber quality traits were evaluated by HVI spectrum: 2.5 % fiber span length (FL, mm), strength (FS, cN/tex), elongation (FE), micronaire reading (FM), and uniformity ratio (FU).

*: Significant QTL

a: See Table 1 for abbreviations

b,c: The genome-wide and linkage group LOD significance thresholds by permutation test

d: The mean value of genotype

e: Percentage phenotypic variation explained

f,g: a1, a2-the additive (or average allele substitution) effects

h: The overall dominance effects
