# Supplementary material for: Variations and Transmission of QTL Alleles for Yield and Fiber Qualities in Upland Cotton Cultivars Developed in China
Source: PLoS One. 2013 Feb 27;8(2):e57220. doi: 10.1371/journal.pone.0057220 (PMC3584144; doi:10.1371/journal.pone.0057220)
Supplement: Table S2 — Association loci for fiber qualities and yield components. (DOC) [file pone.0057220.s004.doc]

**Table S2** Association loci for fiber qualities and yield components

| Traits | Loci | Traits | Loci |
| --- | --- | --- | --- |
| No. fruit branches (PB) | 15 | 2.5% Fiber span length (FL,mm) | 14 |
| Boll weight (BW, g) | 20 | Fiber strength (FS, cN/tex) | 29 |
| Lint percentage (%, LP) | 45 | Micronaire(FM) | 26 |
| No. bolls per plant (NB) | 22 | Uniformity ratio (FU) | 9 |
