# Supplementary material for: Variations and Transmission of QTL Alleles for Yield and Fiber Qualities in Upland Cotton Cultivars Developed in China
Source: PLoS One. 2013 Feb 27;8(2):e57220. doi: 10.1371/journal.pone.0057220 (PMC3584144; doi:10.1371/journal.pone.0057220)
Supplement: Table S3 — Phenotypic effect of QTL alleles significantly associated with traits. (DOC) [file pone.0057220.s005.doc]

**Table s3** Phenotypic effect of QTL alleles significantly associated with traits

| Traits ^a^ | QTL Allele ^b^ | Phenotypic effect a_i_ | Typical cultivars | Traits ^a^ | QTL Allele ^b^ | Phenotypic effect a_i_ | Typical cultivars |
| --- | --- | --- | --- | --- | --- | --- | --- |
| PB | NAU3419-1 | -0.15 | Lumian 5 | NB | NAU3084-1 | 0.24 | Sumian 16 |
|  | NAU3419-2 | 0.21 | Jimian 12 |  | NAU3084-2 | -2.20 | DPL16 |
|  | BNL3792-1 | -0.06 | Ejing 92 | LI(g) | NAU1254-1 | -0.29 | Emian 14 |
|  | BNL3792-2 | 0.20 | Xuzhou 1818 |  | NAU1254-2 | 0.31 | Lumian 6 |
|  | BNL3792-3 | 2.04 | Shanmian 1 |  | NAU3398-1 | 0.02 | DPL16 |
|  | NAU2354-1 | -0.05 | Ejing 92 |  | NAU3398-2 | -0.23 | Simian 2 |
|  | NAU2354-2 | 0.61 | Liaomian 4 |  | NAU3398-3 | 0.80 | CRI 34 |
|  | NAU2354-3 | -0.60 | Hua 101 |  | BNL448-1 | -0.01 | DPL16 |
|  | NAU3917-1 | -0.02 | CRI 45 |  | BNL448-2 | 0.15 | CRI 17 |
|  | NAU3917-2 | 2.04 | Shanmian 1 |  | NAU1302-1 | 0.05 | DPL16 |
|  | TMH05-1 | 0.35 | Xuzhou 1818 |  | NAU1302-2 | -0.23 | STV2B |
|  | TMH05-2 | -0.06 | Ejing 92 | SI(g) | JESPR101-1 | -0.26 | CRI 16 |
| PH(cm) | JESPR232-1 | 0.58 | Esha 28 |  | JESPR101-2 | 0.65 | 57-681 |
|  | JESPR232-2 | -18.30 | CRI 16 |  | JESPR101-3 | -0.14 | Sumian 3 |
|  | JESPR232-3 | -0.79 | Shanmian 1 |  | JESPR101-4 | 0.83 | Shan 1155 |
|  | NAU980-1 | 0.13 | Sumian 2 |  | JESPR101-5 | 2.44 | Yumian 5 |
|  | NAU980-2 | -6.27 | Jingsimian |  | JESPR101-6 | -0.19 | Sumian 12 |
|  | NAU980-3 | 6.25 | Ejing 1 |  | NAU1151-1 | -0.25 | Sumian 12 |
|  | NAU980-4 | -0.98 | Xiangmian 10 |  | NAU1151-2 | 0.66 | CRI 19 |
|  | BNL2646-1 | 0.46 | Sumian 2 |  | JESPR274-1 | 0.18 | TM-1 |
|  | BNL2646-2 | -6.98 | CRI 41 |  | JESPR274-2 | -0.44 | Shiduan 5 |
|  | BNL2646-3 | -2.95 | Lumian 5 |  | JESPR274-3 | -0.14 | Sumian 12 |
|  | JESPR227-1 | 0.23 | Sumian 2 |  | NAU4921-1 | -0.09 | Sumian 12 |
|  | JESPR227-2 | -18.30 | CRI 16 |  | NAU4921-2 | 1.36 | Jingsimian |
|  | NAU5091-1 | -0.49 | Sumian 2 |  | JESPR135-1 | -0.12 | Sumian 3 |
|  | NAU5091-2 | 5.10 | XiangSC-24 |  | JESPR135-2 | 1.83 | Jingsimian |
| LP(%) | NAU3700-1 | 0.57 | CRI 16 |  |  |  |  |
|  | NAU3700-2 | -3.68 | Foster 6 | FL(mm) | NAU1048-1 | 0.20 | Sumian 6 |
|  | NAU3700-3 | -4.27 | Jingsimian |  | NAU1048-2 | -0.09 | Lumian 2 |
|  | BNL448-1 | -0.34 | Sumian 3 |  | NAU4921-1 | -0.04 | CRI 35 |
|  | BNL448-2 | 5.17 | 8891 |  | NAU4921-2 | 0.62 | DPL 16 |
|  | BNL3103-1 | 0.19 | CRI 16 | FS(cN/tex) | NAU422-1 | -0.38 | King |
|  | BNL3103-2 | -7.52 | Shanmian 1 |  | NAU422-2 | 0.07 | Lumian 4 |
|  | NAU1302-1 | 0.49 | CRI 16 |  | NAU2156-1 | -0.06 | Sumian 16 |
|  | NAU1302-2 | -2.31 | Sumian 1 |  | NAU2156-2 | 1.80 | CRI 4133 |
|  | NAU3917-1 | 0.15 | CRI 16 |  | NAU2156-3 | -0.94 | 52-128 |
|  | NAU3917-2 | -11.49 | Shanmian 1 |  | cgr5510-1 | -0.69 | CRI 15 |
|  | NAU2697-1 | 0.71 | CRI 16 |  | cgr5510-2 | 0.12 | Shan 6192 |
|  | NAU2697-2 | -2.01 | CRI 12 | FM | NAU1162-1 | 0.07 | Sumian 9 |
|  | NAU5166-1 | 0.06 | CRI 16 |  | NAU1162-2 | -0.08 | Daihongdai |
|  | NAU5166-2 | 6.48 | Simian 3 |  | NAU1162-3 | -0.29 | Emian 14 |
|  | NAU5166-3 | -11.49 | Shanmian 1 |  | NAU3053-1 | -0.03 | Daihongdai |
|  | NAU3398-1 | -0.36 | Sumian 2 |  | NAU3053-2 | 0.24 | XiangSC-24 |
|  | NAU3398-2 | 1.88 | Ejing 1 |  | NAU3053-3 | 0.55 | Lumian 5 |
|  | NAU3398-3 | 8.26 | Simian 4 | FU | NAU3016-1 | 0.04 | Simian 3 |
| BW(g) | NAU3269-1 | -0.13 | Sumian 12 | (%) | NAU3016-2 | -0.61 | Shanmian 4 |
|  | NAU3269-2 | 0.17 | CRI 34 |  | NAU3061-1 | 0.09 | CRI 3 |
|  | BNL3280-1 | 0.04 | CRI 12 |  | NAU3061-2 | -0.35 | CRI 15 |
|  | BNL3280-2 | -0.28 | Lumian 2 |  | NAU980-1 | 0.01 | CRI 35 |
|  | BNL3280-3 | 0.11 | Keyi 2 |  | NAU980-2 | 0.03 | Shanmian 1 |
|  | BNL3280-4 | -1.26 | Yanmian 1 |  | NAU980-3 | 0.33 | 8891 |
| NB | JESPR101-1 | 0.72 | Shanmian 4 |  | NAU980-4 | -2.01 | Xiangmian10 |
|  | JESPR101-2 | -1.80 | Foster 6 |  | cgr5510-1 | -0.43 | Emian14 |
|  | JESPR101-3 | 0.12 | Sumian 16 |  | cgr5510-2 | 0.08 | CRI3 |
|  | JESPR101-4 | -0.61 | 86-6 | FE | NAU2156-1 | 0.03 | CRI 41 |
|  | JESPR101-5 | -4.79 | 57-681 | (%) | NAU2156-2 | -0.91 | CRI 17 |
|  | JESPR101-6 | -0.94 | Daihongdai |  | NAU2156-3 | 0.41 | 52-128 |
|  | BNL3590-1 | 0.55 | Sumian 6 |  | BNL1681-1 | 0.06 | Lumian 5 |
|  | BNL3590-2 | -2.30 | Foster 6 |  | BNL1681-2 | -0.18 | TM-1 |
|  | BNL3590-3 | -1.40 | Daihongdai |  |  |  |  |
|  | NAU3700-1 | 0.30 | Sumian 6 |  |  |  |  |
|  | NAU3700-2 | -2.52 | Liaomian 4 |  |  |  |  |
|  | NAU3700-3 | -1.32 | Keyi 2 |  |  |  |  |
